# Association of Glycemic Status with Disability, Relapse Activity, Inflammatory Markers, and Metabolic Profile in Patients with Multiple Sclerosis

**DOI:** 10.3390/jcm15155960

**Published:** 2026-07-30

**Authors:** Soner Yeşilyurt, Furkan Talha Tokdemir, Mehmet Tayfur, Burcu Altunrende, Hafize Uzun

**Affiliations:** 1 Department of Internal Medicine, Taksim Training and Research Hospital, University of Health Sciences, Istanbul 34764, Türkiye; 2 Department of Neurology, Taksim Training and Research Hospital, University of Health Sciences, Istanbul 34433, Türkiye; doctorftt@gmail.com (F.T.T.); burcunoro@gmail.com (B.A.); 3 Department of Internal Medicine, Ümraniye Training and Research Hospital, University of Health Sciences, Istanbul 34764, Türkiye; drmtayfur@gmail.com; 4 Department of Biochemistry, Faculty of Medicine, Istanbul Atlas University, Istanbul 34408, Türkiye; hafize.uzun@atlas.edu.tr

**Keywords:** multiple sclerosis, glycemic status, diabetes mellitus, prediabetes, HbA1c, Expanded Disability Status Scale, annualized relapse rate, systemic inflammation, metabolic syndrome, triglyceride–glucose index

## Abstract

**Background/Objectives:** Metabolic dysregulation has emerged as a potential modifier of disease activity and disability in multiple sclerosis (MS). However, the relationship between glycemic status and clinical outcomes in patients with MS remains incompletely understood. This study aimed to investigate the associations of glycemic regulation with disease severity, relapse activity, inflammatory markers, and metabolic characteristics in patients with MS. **Methods:** In this retrospective single-center observational study, 455 patients with MS aged 18–65 years were included. Patients were categorized into normoglycemia (n = 241), prediabetes (n = 98), and diabetes (n = 116) according to American Diabetes Association criteria and documented diabetes diagnosis. Demographic characteristics, Expanded Disability Status Scale (EDSS) scores, annualized relapse rate (ARR), disease-modifying therapy (DMT), hematological indices, inflammatory markers, and metabolic parameters were retrieved from electronic medical records. Associations between glycemic status and clinical outcomes were evaluated using group comparisons, Spearman correlation analyses, and multivariable linear regression models. **Results:** Significant differences were observed among glycemic groups for age, disease duration, EDSS score, ARR, MS phenotype, platelet count, C-reactive protein (CRP), erythrocyte sedimentation rate (ESR), fasting plasma glucose (FPG), glycated hemoglobin (HbA1c), lipid parameters, estimated glomerular filtration rate, alanine aminotransferase, gamma-glutamyl transferase, triglyceride–glucose (TyG) index, and atherogenic index of plasma (all *p* < 0.05). HbA1c, FPG, TyG index, lipid parameters, CRP, and ESR were positively correlated with EDSS scores (all *p* < 0.05). In contrast, these markers showed inverse correlations with ARR. However, after adjustment for demographic and clinical variables, including age, sex, disease duration, MS phenotype, and disease-modifying therapy (DMT), neither prediabetes nor diabetes remained independently associated with disability, as measured by the EDSS score, or with ARR. Progressive MS phenotype, older age, and longer disease duration were independently associated with higher disability, whereas age, disease duration, and progressive phenotype were independently associated with lower ARR. **Conclusions:** Impaired glycemic status is associated with greater disability, unfavorable metabolic profiles, and increased systemic inflammation in patients with MS. Nevertheless, glycemic status was not independently associated with disability or relapse activity after adjustment for major clinical confounders. These findings suggest that metabolic dysregulation accompanies more severe clinical characteristics but may not independently drive disease progression in MS.

## 1. Introduction

Multiple sclerosis (MS) is a chronic immune-mediated inflammatory disease of the central nervous system (CNS) characterized by demyelination, axonal injury, and progressive neurodegeneration. It is one of the leading causes of non-traumatic neurological disability among young adults worldwide. Despite major advances in disease-modifying therapies, considerable heterogeneity remains in disease progression, relapse frequency, and disability accumulation, highlighting the importance of identifying modifiable clinical factors that may influence disease outcomes [1,2].

Metabolic disorders have recently attracted increasing attention as potential modifiers of neuroinflammatory diseases. Diabetes mellitus (DM), particularly type 2 diabetes mellitus (T2DM), is characterized by chronic hyperglycemia, systemic inflammation, oxidative stress, endothelial dysfunction, and immune dysregulation. These mechanisms overlap with several pathological pathways involved in MS, suggesting that impaired glucose metabolism may contribute to disease activity and neurological deterioration [2,3]. Hyperglycemia has been implicated in mechanisms that may promote neuroinflammation, including increased production of pro-inflammatory cytokines, mitochondrial dysfunction, and disruption of the blood–brain barrier (BBB) [4].

The coexistence of MS and diabetes has become increasingly common owing to the rising global prevalence of metabolic diseases. Epidemiological studies have suggested that diabetes may adversely influence the clinical course of MS through increased disability, fatigue, vascular comorbidity, and impaired physical function [3]. Moreover, high-dose corticosteroids, the standard treatment for acute MS relapses, frequently induce transient hyperglycemia, which may complicate clinical management and potentially affect recovery following relapse [5].

Recent evidence indicates that T2DM may increase the risk of multiple sclerosis, whereas current data do not support a significantly increased risk of diabetes among patients with established MS. A comprehensive meta-analysis reported that individuals with diabetes, particularly T2DM, have an increased risk of developing MS, whereas current evidence does not support a significantly increased risk of diabetes among patients with established MS [6]. Nevertheless, the biological interactions between glucose metabolism and disease progression remain incompletely understood, and previous studies have largely focused on disease incidence rather than clinical outcomes after MS diagnosis.

Beyond conventional measures of glycemic control, composite metabolic and inflammatory biomarkers have recently emerged as practical tools for assessing cardiometabolic risk and systemic inflammation. The triglyceride–glucose (TyG) index and the atherogenic index of plasma (AIP) have been recognized as surrogate markers of insulin resistance and atherogenic dyslipidemia, respectively, whereas hematological inflammatory indices and acute-phase reactants such as C-reactive protein (CRP) and erythrocyte sedimentation rate (ESR) provide readily available indicators of systemic inflammatory activity [7,8]. Although these biomarkers have shown prognostic value in various metabolic and cardiovascular disorders, their clinical significance in relation to glycemic status and disease severity in patients with MS remains insufficiently investigated.

Importantly, only limited evidence has directly evaluated whether glycemic regulation is associated with clinically relevant outcomes such as relapse frequency, disability progression assessed by the Expanded Disability Status Scale (EDSS), morbidity, or mortality in patients with MS. Therefore, the impact of chronic glucose dysregulation on disease severity and prognosis remains unclear.

The present study aimed to evaluate the association of glycemic status with disability, as measured by the EDSS score, annualized relapse rate (ARR), inflammatory markers, and metabolic indices, including the TyG index and the AIP, in patients with MS. By clarifying these relationships, this study sought to improve the understanding of the clinical significance of glycemic dysregulation and to support comprehensive metabolic assessment in the routine management of patients with MS.

## 2. Materials and Methods

### 2.1. Study Design and Participants

The study protocol was approved by the Non-Interventional Clinical Research Ethics Committee of Taksim Training and Research Hospital (Approval No: E-6290176-663.05-269205258; Date: 21 February 2025). Owing to the retrospective design of the study and the use of anonymized hospital records, the requirement for informed consent was waived by the Ethics Committee. The study was conducted in accordance with the principles of the Declaration of Helsinki.

This retrospective observational study was conducted at the Department of Neurology, Taksim Training and Research Hospital, Istanbul, Türkiye. Electronic medical records of consecutive patients diagnosed with MS between January 2020 and January 2025 were retrospectively reviewed using the hospital electronic medical record system.

An a priori sample size calculation indicated that a minimum of 130 patients was required to detect a correlation of approximately 0.25 between glycemic markers and disability (EDSS score) with 80% power at a two-sided α of 0.05; all eligible consecutive patients meeting the study criteria were included. Patients aged between 18 and 65 years who had been diagnosed with MS according to the 2017 revised McDonald criteria and had been followed for at least six months were eligible for inclusion [9]. Patients with incomplete clinical or laboratory data were excluded from the study. The diabetes group consisted exclusively of patients with type 2 diabetes mellitus identified from the electronic medical records, whereas patients with T1DM were excluded because of their distinct autoimmune pathophysiology and clinical characteristics.

The exclusion criteria were: (i) corticosteroid treatment within the previous three months, (ii) acute infection at the time of laboratory assessment, (iii) active malignancy, (iv) chronic organ failure (including advanced renal, hepatic, or heart failure), and (v) concomitant systemic inflammatory or autoimmune diseases other than MS.

### 2.2. Classification of Glycemic Status

Patients were classified into normoglycemia, prediabetes, and diabetes according to the American Diabetes Association (ADA) criteria [10]. Prediabetes was defined as a fasting plasma glucose (FPG) concentration of 100–125 mg/dL and/or a glycated hemoglobin (HbA1c) level of 5.7–6.4%. Diabetes was defined as an FPG concentration ≥126 mg/dL and/or an HbA1c level ≥6.5%. Patients with a documented physician diagnosis of T2DM or receiving glucose-lowering medications prescribed for T2DM, including insulin therapy, were also classified as having diabetes.

Glycemic status was determined using the documented diagnosis in the electronic medical records together with the corresponding FPG and HbA1c measurements obtained during the same clinical assessment in which the complete blood count (CBC) and other laboratory parameters included in this study were collected. Thus, the classification of glycemic status corresponded to the same time period as the laboratory measurements analyzed in this study.

### 2.3. Data Collection

Clinical and demographic information was retrospectively extracted from the electronic medical records of all eligible patients. The collected variables included age, sex, disease duration, previous and current disease-modifying therapies (DMTs), EDSS score, and the complete documented relapse history.

Current DMTs were classified according to therapeutic efficacy as low-efficacy therapies (interferon preparations, glatiramer acetate, teriflunomide, and dimethyl fumarate), high-efficacy therapies (alemtuzumab, cladribine, natalizumab, ocrelizumab, ofatumumab, and sphingosine-1-phosphate receptor modulators), and immune reconstitution therapy with autologous hematopoietic stem cell transplantation (AHSCT). The cumulative duration of DMT exposure was calculated from the initiation of the first DMT until the date of laboratory evaluation. Disability was assessed using the EDSS, as originally described by Kurtzke [11].

Patients were classified according to the clinical phenotype of MS as relapsing–remitting MS (RRMS), secondary progressive MS (SPMS), or primary progressive MS (PPMS). Only patients with a confirmed diagnosis based on the 2017 revised McDonald criteria were included [9]. Patients with clinically isolated syndrome (CIS), radiologically isolated syndrome (RIS), or an unclassified MS phenotype were excluded from the analyses.

Annualized relapse rate (ARR) was calculated from the documented relapse history recorded in the electronic medical records over the entire disease course up to the laboratory assessment. ARR was defined as the total number of confirmed clinical relapses since disease onset divided by disease duration in years, rather than being extrapolated from the three months preceding laboratory evaluation.

Routine laboratory measurements, including hematological indices, serum lipid profile, and biochemical parameters, were extracted from the hospital electronic medical records. Peripheral venous blood samples were collected during the morning after at least 12 h of overnight fasting according to the institution’s standard laboratory protocol. All laboratory parameters, including the CBC, FPG, HbA1c, lipid profile, CRP, and ESR, were obtained from blood samples collected during the same clinical visit. Only laboratory results measured on the same day were included in the analysis.

Inflammatory indices were calculated from routine CBC parameters obtained at the time of blood sampling. The neutrophil-to-lymphocyte ratio (NLR) was calculated by dividing the absolute neutrophil count by the absolute lymphocyte count, the monocyte-to-lymphocyte ratio (MLR) by dividing the absolute monocyte count by the absolute lymphocyte count, and the platelet-to-lymphocyte ratio (PLR) by dividing the platelet count by the absolute lymphocyte count. The systemic immune–inflammation index (SII) was calculated using the formula: platelet count × neutrophil count/lymphocyte count [12] and the systemic inflammation response index (SIRI) = neutrophil count × monocyte count/lymphocyte count [13].

The AIP is derived from the formula: log10 (TG [mg/dL]/HDL − C [mg/dL]) [14].

The TyG index is calculated as ln (TG [mg/dL] × FPG [mg/dL]/2) [15].

### 2.4. Statistical Analysis

Statistical analysis was performed using IBM SPSS Statistics version 31.0 (IBM Corp., Armonk, NY, USA) and Python version 3.13.1 (Python Software Foundation, Wilmington, DE, USA). Continuous variables were evaluated for normality and are presented as mean ± standard deviation or median [interquartile range], as appropriate. Categorical variables are reported as n (%). Comparisons among the normoglycemia, prediabetes, and diabetes groups were performed using one-way ANOVA, Welch ANOVA, or the Kruskal–Wallis test. Significant omnibus tests were followed by Tukey, Bonferroni-adjusted Welch, or Dunn–Bonferroni post hoc comparisons, as appropriate. Categorical variables were compared using Pearson’s chi-square test or exact tests for sparse contingency tables. Effect sizes were reported as eta squared (η^2^), epsilon squared (ε^2^), Cramér’s V, rank-biserial correlation, or Hedges’ g, according to the statistical test used.

Spearman’s correlation analysis was used to examine associations between laboratory markers and EDSS score, ARR, and disease duration. ARR was calculated as the cumulative number of documented relapses divided by disease duration in years. To account for multiple comparisons, Benjamini–Hochberg false discovery rate correction was applied within related analysis families, and adjusted q values are reported alongside *p* values.

Separate multivariable linear regression models were constructed for EDSS and ARR. Covariates were selected a priori and included glycemic status, age, sex, disease duration, MS phenotype, and DMT step. Multicollinearity was assessed using tolerance and variance inflation factor values. Because heteroscedasticity was detected, HC3 robust standard errors were used. Results are presented as unstandardized B and standardized β coefficients with 95% confidence intervals.

Sensitivity analyses separated ICD-coded and laboratory-defined diabetes, excluded laboratory-defined cases, and replaced DMT step with a binary low- versus high-efficacy treatment category. Missing secondary laboratory measurements were handled using available-case analysis; no mean imputation was performed. All tests were two-sided, with *p* < 0.05 or FDR-adjusted q < 0.05 considered statistically significant.

## 3. Results

### 3.1. Patient Selection and Glycemic Status Distribution

**Figure 1:**
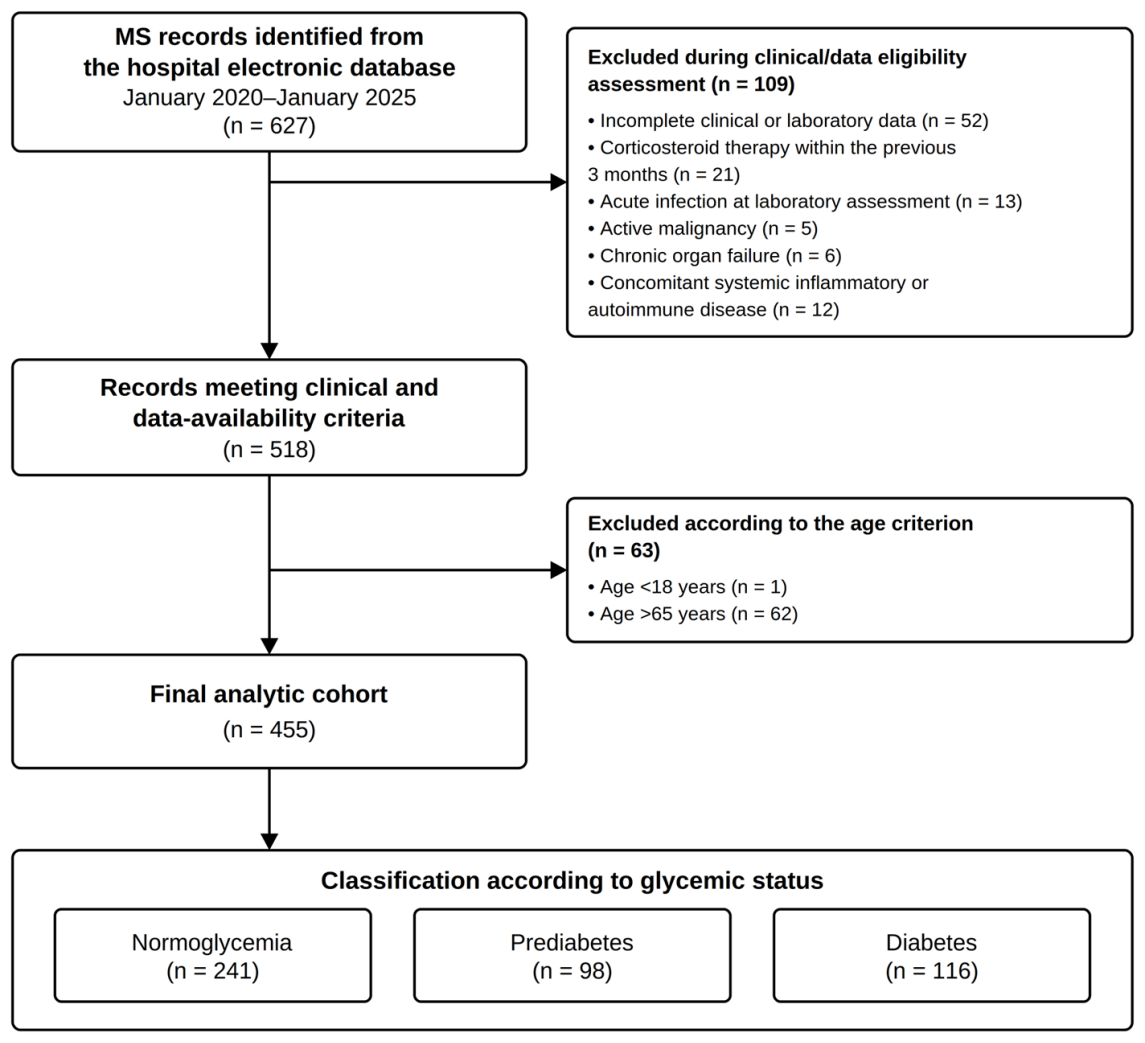
Flow diagram of the patient selection process and reasons for exclusion.

A total of 627 patients with a diagnosis of MS were identified from the hospital database and screened for eligibility. During the clinical and data-eligibility assessment, 109 patients were excluded because of incomplete clinical or laboratory data (n = 52), corticosteroid treatment within the previous 3 months (n = 21), acute infection at the time of laboratory assessment (n = 13), active malignancy (n = 5), chronic organ failure (n = 6), or concomitant systemic inflammatory or autoimmune diseases other than MS (n = 12), leaving 518 records that met the clinical and data-availability criteria. Patients with concomitant systemic inflammatory or autoimmune diseases other than MS were excluded because these conditions may independently influence inflammatory biomarkers, glucose metabolism, and clinical outcomes, potentially confounding the associations investigated in this study. Of these 518 records, 63 were subsequently excluded because they did not meet the predefined age criterion (18–65 years), including one patient younger than 18 years and 62 patients older than 65 years. The final analytic cohort comprised 455 patients, including 241 (53.0%) with normoglycemia, 98 (21.5%) with prediabetes, and 116 (25.5%) with diabetes. The patient selection process and detailed reasons for exclusion are presented in Figure 1.

### 3.2. Demographic and Clinical Characteristics According to Glycemic Status

The demographic and clinical characteristics of the study population according to glycemic status are presented in Table 1. Age differed significantly among the three groups (*p* < 0.001, ε^2^ = 0.083). Patients with prediabetes had the highest median age, followed by those with diabetes and normoglycemia. Disease duration also differed significantly across glycemic status groups (*p* = 0.005, ε^2^ = 0.019), with the longest median disease duration observed in the prediabetes group.

EDSS scores differed significantly among the groups (*p* < 0.001, ε^2^ = 0.039). Although the median EDSS score was 2.00 in all three groups, the interquartile range was wider in the prediabetes group. EDSS category distribution also differed significantly according to glycemic status (*p* < 0.001, Cramer’s V = 0.154), with mild disability being most frequent in the normoglycemia group and moderate disability being more frequent among patients with prediabetes and diabetes. Because the number of patients in the advanced EDSS category was small, this categorical finding should be interpreted cautiously.

The total number of relapses did not differ significantly among the groups (*p* = 0.314, ε^2^ = 0.001). However, ARR differed significantly according to glycemic status (*p* = 0.005, ε^2^ = 0.019). Sex distribution was not significantly different among the groups (*p* = 0.156, Cramer’s V = 0.090). MS phenotype distribution differed significantly across glycemic status groups (*p* < 0.001, Cramer’s V = 0.149), with a relatively higher proportion of progressive MS phenotypes in the prediabetes group. DMT treatment step did not differ significantly among the groups (*p* = 0.323, Cramer’s V = 0.088).

### 3.3. Hematological and Inflammatory Markers According to Glycemic Status

Hematological and inflammatory parameters are summarized in Table 2. Platelet count differed significantly among glycemic status groups (*p* = 0.011, ε^2^ = 0.016), with higher median values in the prediabetes and diabetes groups than in the normoglycemia group. CRP levels also differed significantly among the groups (*p* < 0.001, ε^2^ = 0.030), with the highest median value observed in the prediabetes group. Similarly, ESR differed significantly by glycemic status (*p* = 0.002, ε^2^ = 0.022).

No significant differences were observed among the groups for white blood cell count, neutrophil count, lymphocyte count, monocyte count, NLR, MLR, PLR, or systemic immune-inflammation index. Overall, glycemic status was associated with selected inflammatory markers, particularly platelet count, CRP, and ESR, although the corresponding effect sizes were small.

### 3.4. Metabolic, Lipid, Renal, and Hepatic Profile According to Glycemic Status

Metabolic, lipid, renal, and hepatic parameters are presented in Table 3. As expected, fasting glucose and HbA1c differed significantly among the groups (both *p* < 0.001), with the largest effect sizes observed for HbA1c (ε^2^ = 0.310) and glucose (ε^2^ = 0.178). The highest median fasting glucose and HbA1c values were observed in the prediabetes group, whereas the diabetes group showed intermediate median values. This reflects the composition of the diabetes group, which by definition included patients receiving glucose-lowering medication: 96 of 116 patients (82.8%) were ICD-coded and under clinical management, whereas 20 (17.2%) were laboratory-defined. Median HbA1c was 5.40% [5.20–5.80] in the ICD-coded subgroup versus 6.80% [5.75–8.50] in the laboratory-defined subgroup, with CRP correspondingly lower (1.67 [0.61–3.41] vs. 4.63 [1.70–7.23] mg/L, *p* = 0.006; Supplementary Table S4).

Lipid parameters also differed significantly according to glycemic status. Total cholesterol (*p* = 0.001, ε^2^ = 0.028), LDL cholesterol (*p* < 0.001, ε^2^ = 0.032), HDL cholesterol (*p* = 0.034, ε^2^ = 0.011), and triglycerides (*p* < 0.001, ε^2^ = 0.038) showed significant between-group differences. AIP differed significantly among the groups (*p* < 0.001, η^2^ = 0.048), with higher values in the prediabetes and diabetes groups than in the normoglycemia group. TyG index also differed significantly across groups (*p* < 0.001, η^2^ = 0.091), indicating greater metabolic burden in patients with impaired glycemic status.

Renal function, assessed using estimated glomerular filtration rate (eGFR), differed significantly among the groups (*p* < 0.001, ε^2^ = 0.032), whereas creatinine levels did not differ significantly (*p* = 0.491, ε^2^ = 0.000). Among hepatic markers, ALT (*p* = 0.001, ε^2^ = 0.029) and GGT (*p* < 0.001, ε^2^ = 0.045) differed significantly according to glycemic status, while AST did not show a significant difference (*p* = 0.370, ε^2^ = 0.000).

The extended laboratory profile is shown in Supplementary Table S2. In this extended analysis, several additional laboratory parameters differed significantly among glycemic status groups, including MCV, MCH, RDW, plateletcrit, BUN, urea, chloride, ALP, CK, albumin, iron, UIBC, folate, and free T4. However, most of these differences had small effect sizes and should be considered supportive rather than primary findings.

### 3.5. Effect-Size Profile of Between-Group Differences

**Figure 2:**
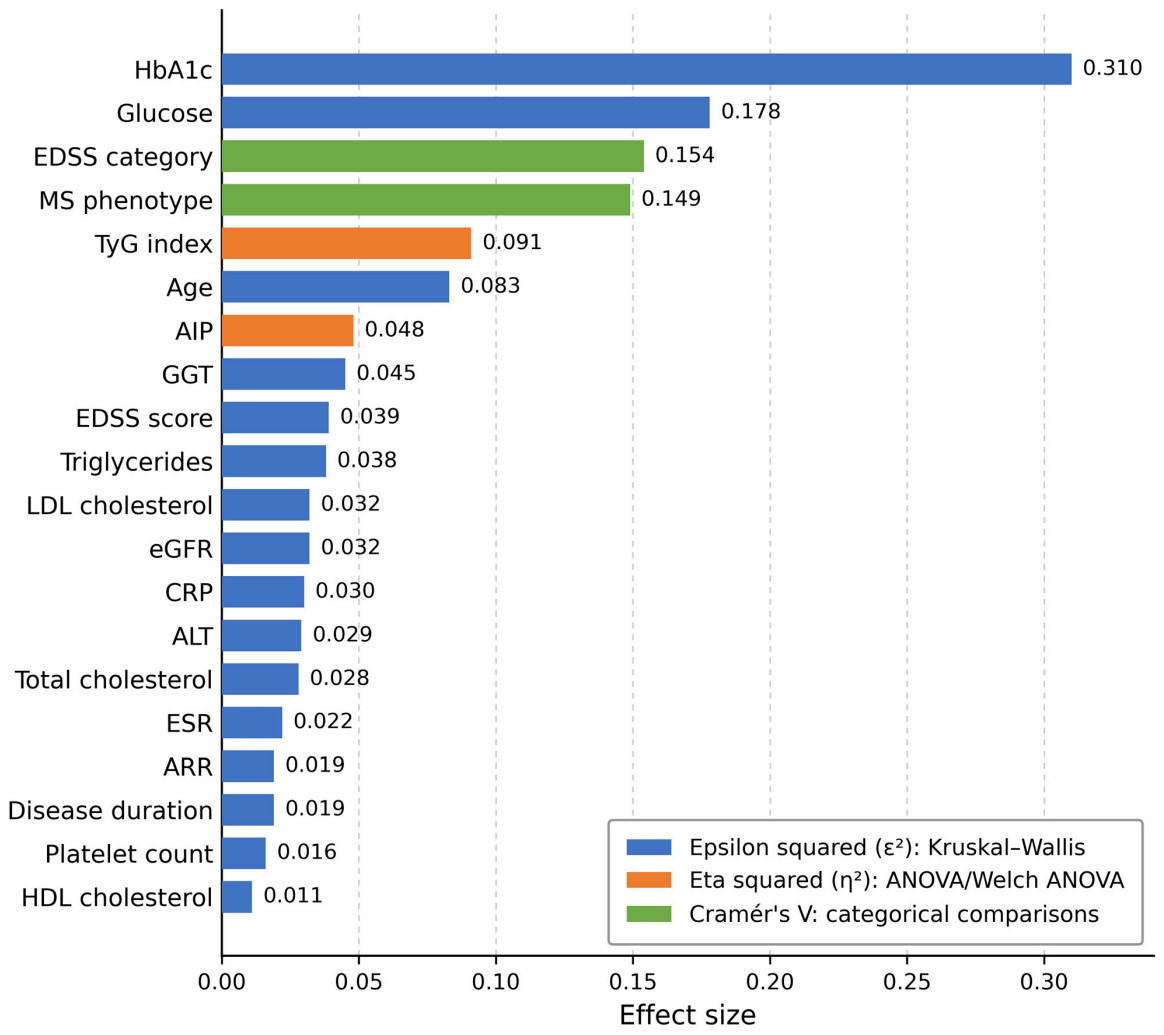
Effect sizes of variables significantly associated with glycemic status. Blue bars indicate ε^2^ (Kruskal–Wallis test), orange bars indicate η^2^ (ANOVA/Welch ANOVA), and green bars indicate Cramér’s V (categorical variables). Because these effect size measures are derived from different statistical methods, they should not be interpreted as directly comparable.

The overall pattern of effect sizes for variables differing by glycemic status is summarized in Figure 2. The largest effect sizes were observed for HbA1c and glucose, followed by EDSS category, MS phenotype, TyG index, and age. Smaller effect sizes were observed for hepatic, lipid, inflammatory, renal, and relapse-related parameters. Because Figure 2 includes effect sizes derived from different statistical tests, including ε^2^, η^2^, and Cramer’s V, the figure should be interpreted as a descriptive overview of the magnitude of between-group differences rather than as a direct comparison across identical effect-size metrics.

### 3.6. Correlations Between Metabolic, Inflammatory Markers and Clinical MS Outcomes

Spearman correlation analyses between glycemic, metabolic, lipid, and inflammatory markers and clinical MS outcomes are presented in Table 4. EDSS showed significant positive correlations with FPG concentrations (ρ = 0.189, *p* < 0.001), HbA1c (ρ = 0.249, *p* < 0.001), TyG index (ρ = 0.227, *p* < 0.001), AIP (ρ = 0.140, *p* = 0.003), total cholesterol (ρ = 0.211, *p* < 0.001), LDL cholesterol (ρ = 0.181, *p* < 0.001), triglycerides (ρ = 0.187, *p* < 0.001), CRP (ρ = 0.163, *p* < 0.001), and ESR (ρ = 0.177, *p* < 0.001). These findings indicate that higher glycemic, metabolic, lipid, and selected inflammatory marker levels were associated with greater disability burden.

ARR showed significant inverse correlations with FPG concentrations (ρ = −0.198, *p* < 0.001), HbA1c (ρ = −0.264, *p* < 0.001), TyG index (ρ = −0.236, *p* < 0.001), AIP (ρ = −0.168, *p* < 0.001), total cholesterol (ρ = −0.294, *p* < 0.001), LDL cholesterol (ρ = −0.298, *p* < 0.001), triglycerides (ρ = −0.210, *p* < 0.001), CRP (ρ = −0.117, *p* = 0.012), and ESR (ρ = −0.133, *p* = 0.005). Disease duration was positively correlated with glucose, HbA1c, TyG index, AIP, total cholesterol, LDL cholesterol, triglycerides, CRP, and ESR. HDL cholesterol was not significantly correlated with EDSS, ARR, or disease duration. Hematological inflammatory ratios, including NLR, MLR, PLR, and SII, were also not significantly correlated with these clinical outcomes.

Pairwise post hoc analyses are provided in Supplementary Table S1. Prediabetes differed from normoglycemia for age, disease duration, EDSS score, ARR, CRP, ESR, glucose, HbA1c, lipid parameters, eGFR, ALT, GGT, AIP, TyG index, MS phenotype, and EDSS category. Diabetes differed from normoglycemia for age, EDSS score, platelet count, glucose, HbA1c, total cholesterol, LDL cholesterol, triglycerides, ALT, GGT, AIP, TyG index, and EDSS category. In contrast, fewer significant differences were observed between the prediabetes and diabetes groups.

### 3.7. Multivariable Regression Models for EDSS and ARR

**Figure 3:**
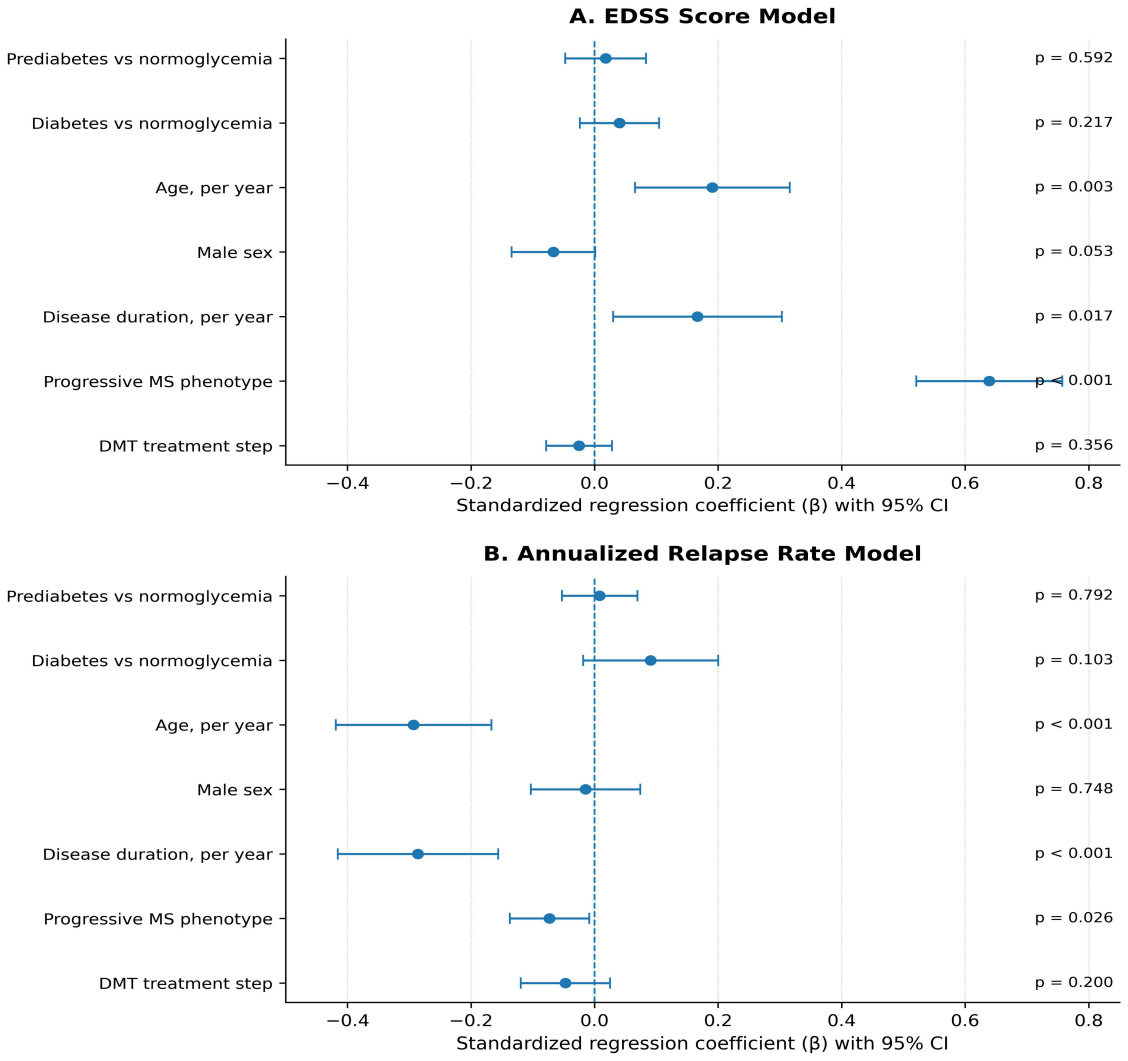
Forest plots of the multivariable regression models for (**A**) Expanded Disability Status Scale (EDSS) and (**B**) annualized relapse rate (ARR). Standardized regression coefficients (β) with 95% confidence intervals are shown for glycemic status, age, sex, disease duration, progressive multiple sclerosis (MS) phenotype, and disease-modifying therapy (DMT) treatment step. The dashed vertical line indicates the null effect (β = 0). *p* values are presented on the right. Regression models were estimated using heteroscedasticity-robust standard errors.

Multivariable regression models evaluating the independent associations of glycemic status and clinical covariates with EDSS and ARR are presented in Table 5. In the EDSS model, prediabetes was not independently associated with disability as measured by the EDSS score compared with normoglycemia (B = 0.039, 95% CI: −0.105 to 0.183, *p* = 0.592). Similarly, diabetes was not independently associated with disability as measured by the EDSS score after adjustment for demographic (age and sex) and clinical covariates (B = 0.084, 95% CI: −0.049 to 0.217, *p* = 0.217). Age (B = 0.015, 95% CI: 0.005 to 0.024, *p* = 0.003), disease duration (B = 0.027, 95% CI: 0.005 to 0.049, *p* = 0.017), and progressive MS phenotype (B = 1.708, 95% CI: 1.392 to 2.024, *p* < 0.001) were independently associated with higher EDSS scores. Progressive MS phenotype showed the largest standardized effect in the EDSS model (standardized β = 0.639). Male sex showed a borderline association with lower EDSS scores (B = −0.127, 95% CI: −0.256 to 0.002, *p* = 0.053), whereas DMT treatment step was not independently associated with disability as measured by the EDSS score (*p* = 0.356). The EDSS model explained 64.5% of the variance in EDSS scores (R^2^ = 0.645, adjusted R^2^ = 0.639). These EDSS model estimates are visualized in Figure 3.

In the ARR model, prediabetes was not independently associated with ARR compared with normoglycemia (B = 0.002, 95% CI: −0.016 to 0.021, *p* = 0.792). Diabetes also did not reach statistical significance as an independent predictor of ARR (B = 0.026, 95% CI: −0.005 to 0.057, *p* = 0.103). Age (B = −0.003, 95% CI: −0.004 to −0.002, *p* < 0.001), disease duration (B = −0.006, 95% CI: −0.009 to −0.003, *p* < 0.001), and progressive MS phenotype (B = −0.027, 95% CI: −0.050 to −0.003, *p* = 0.026) were independently associated with lower ARR. DMT treatment step was not independently associated with ARR (*p* = 0.200). The ARR model explained 30.8% of the variance in ARR (R^2^ = 0.308, adjusted R^2^ = 0.297).

Model diagnostics showed no evidence of substantial multicollinearity, with all variance inflation factor values below 5 and all tolerance values above 0.20. The Durbin-Watson statistics were 1.942 for the EDSS model and 1.997 for the ARR model, suggesting no major autocorrelation issue. The Breusch-Pagan test indicated heteroscedasticity in both models; therefore, heteroscedasticity-robust standard errors were used.

## 4. Discussion

The present study provides a comprehensive evaluation of the relationship between glycemic status and clinical characteristics in patients with multiple sclerosis. Patients with prediabetes and diabetes exhibited greater disability, reflected by higher EDSS scores, together with a less favorable metabolic profile and increased systemic inflammatory burden, as evidenced by higher CRP and ESR levels, compared with normoglycemic patients. Furthermore, glycemic and metabolic markers, including fasting glucose, HbA1c, the TyG index, and lipid parameters, were significantly correlated with disability and relapse activity. However, these associations did not persist after adjustment for demographic (age and sex) and clinical covariates. In multivariable analyses, glycemic status was not independently associated with disability as measured by the EDSS score or ARR, whereas older age, longer disease duration, and progressive MS phenotype emerged as the principal determinants of disability and relapse activity. Collectively, these findings suggest that glycemic dysregulation accompanies a more adverse metabolic and inflammatory profile and greater clinical disease burden in patients with MS, but its apparent relationship with disability is largely explained by established clinical determinants rather than representing an independent predictor of disease severity.

Our findings indicate that impaired glycemic status is accompanied by distinct differences in demographic and clinical characteristics among patients with MS. Patients with prediabetes were older, had a longer disease duration, and exhibited higher disability levels, as reflected by EDSS scores and disability categories, than normoglycemic patients. In addition, progressive MS phenotypes were more frequent among individuals with prediabetes, whereas treatment intensity with disease-modifying therapies did not differ according to glycemic status. These findings are generally consistent with previous studies reporting that metabolic comorbidities, particularly T2DM, are associated with greater physical disability and a more unfavorable clinical course in MS [3,16,17,18]. Maric et al. [16] demonstrated that diabetes contributes to disability progression measured by EDSS independently of cardiovascular comorbidities, emphasizing the adverse clinical impact of metabolic disorders in MS. Similarly, Ciampi et al. [17] reported that patients with MS and metabolic comorbidities exhibited greater physical disability, particularly among progressive disease phenotypes. Furthermore, a recent systematic review by Giannopapas et al. [3] concluded that the coexistence of MS and T2DM is associated with increased disability, fatigue, reduced functional capacity, and poorer quality of life, despite the relatively low prevalence of diabetes in the MS population. In addition, Oliveira et al. [18] suggested that insulin resistance and metabolic dysfunction may contribute to disability progression through chronic inflammation and oxidative stress, supporting a biological link between impaired glucose metabolism and neurological impairment in MS. Although we also observed significant differences in EDSS and relapse activity across glycemic groups, these between-group differences should be interpreted in the context of the older age, longer disease duration, and higher proportion of progressive MS phenotypes among patients with impaired glycemic status, all of which are well-established determinants of disability accumulation in MS. These observations support the hypothesis that glycemic dysregulation may represent a marker of a more adverse clinical phenotype rather than an independent determinant of disease severity.

In the present study, impaired glycemic status was associated with higher platelet counts and increased levels of the conventional inflammatory markers CRP and ESR, whereas leukocyte counts and CBC-derived inflammatory indices, including NLR, MLR, PLR, and SII, did not differ significantly among the glycemic groups. These findings suggest that glycemic dysregulation in patients with MS is accompanied by a state of low-grade systemic inflammation that is more readily reflected by established inflammatory biomarkers than by composite hematological indices. CRP is a widely used marker of systemic inflammation and has been investigated as a potential biomarker of disease activity in MS. However, the available evidence remains inconsistent. While some studies have reported higher CRP levels during clinical relapses or in patients with greater inflammatory disease activity, others have found no significant association between CRP levels and disability progression or long-term clinical outcomes. Therefore, the prognostic value of CRP in MS remains uncertain and warrants further investigation [19,20]. Previous studies have consistently shown that both CRP and ESR are elevated in patients with MS, particularly during periods of increased disease activity, and are associated with enhanced inflammatory burden and disability progression [6,21,22,23,24]. Moreover, chronic low-grade inflammation and oxidative stress are recognized contributors to the pathophysiology of both metabolic disorders and multiple sclerosis. Persistent inflammatory activation, endothelial dysfunction, and oxidative stress may amplify systemic inflammatory responses and contribute to neurodegenerative processes in patients with MS [25,26]. In contrast, the absence of significant differences in NLR, MLR, PLR, and SII is in agreement with reports indicating that these hematological indices are influenced by multiple factors, including disease-modifying therapies, disease stage, and acute inflammatory activity, thereby limiting their ability to discriminate chronic metabolic disturbances in clinically stable MS populations [27,28,29]. The relatively small effect sizes observed for CRP, ESR, and platelet count in our cohort further suggest that although impaired glycemic status is associated with increased systemic inflammation, its contribution to the overall inflammatory burden appears to be modest.

Our study further demonstrated that impaired glycemic status was accompanied by an unfavorable metabolic profile, characterized by higher fasting glucose, HbA1c, atherogenic lipid parameters, TyG index, and AIP. These findings are biologically plausible, as the TyG index is recognized as a reliable surrogate marker of insulin resistance, whereas AIP reflects the combined burden of hypertriglyceridemia and reduced HDL cholesterol and has been associated with increased cardiometabolic risk [18,30,31,32,33]. Although evidence regarding these indices in MS remains limited, insulin resistance and dyslipidemia have been increasingly implicated in disease progression through mechanisms involving chronic low-grade inflammation, oxidative stress, endothelial dysfunction, and mitochondrial impairment [34,35]. Consistent with previous reports, we also observed higher total cholesterol, LDL cholesterol, and triglyceride concentrations among patients with impaired glycemic status. Dyslipidemia has previously been linked to greater disability, accelerated brain atrophy, and worse magnetic resonance imaging outcomes in patients with MS, suggesting that metabolic abnormalities may contribute to neurodegeneration beyond their cardiovascular effects [36]. These lipid changes appear to correlate with BBB dysfunction, suggesting a link between peripheral lipid metabolism and CNS inflammation [35]. In addition, the higher ALT and GGT levels observed in the prediabetes and diabetes groups may indicate underlying metabolic dysfunction, including hepatic insulin resistance and metabolic-associated fatty liver disease, conditions that frequently coexist with impaired glucose metabolism. Collectively, these findings suggest that glycemic dysregulation in MS is accompanied by a broader metabolic disturbance rather than isolated abnormalities in glucose homeostasis. The effect-size profile indicates that the largest between-group differences were observed for markers directly reflecting glycemic regulation, whereas clinical and inflammatory variables demonstrated only small-to-moderate effect sizes. This pattern suggests that although impaired glycemic status is accompanied by measurable differences in disability and inflammatory burden, these changes are substantially less pronounced than the metabolic alterations defining glycemic status itself.

Correlation analyses further demonstrated that worsening glycemic regulation and metabolic dysfunction were associated with greater neurological disability. EDSS showed significant positive correlations with fasting glucose, HbA1c, TyG index, AIP, atherogenic lipid parameters, CRP, and ESR, suggesting that impaired glucose metabolism, dyslipidemia, and systemic inflammation coexist with increased disability in patients with MS. Similar associations between insulin resistance, adverse lipid profiles, and disability progression have previously been reported, supporting the concept that metabolic dysfunction may contribute to neurodegeneration through chronic inflammation, oxidative stress, and vascular injury [25,26,27,28,29,30,31,32,33,34]. In contrast, ARR was inversely correlated with these metabolic markers. This finding should be interpreted cautiously, as relapse frequency generally declines with increasing age, longer disease duration, and transition to progressive MS phenotypes, despite continued disability accumulation. Therefore, the observed negative correlations are more likely to reflect differences in disease stage than a protective effect of impaired glycemic status. Notably, hematological inflammatory indices, including NLR, MLR, PLR, and SII, were not significantly correlated with disability or relapse activity, suggesting that conventional inflammatory markers such as CRP and ESR may better reflect the chronic inflammatory milieu associated with metabolic dysregulation in clinically stable patients with MS.

Another potential source of residual confounding is the absence of detailed information regarding comorbid conditions. Hypertension, cardiovascular disease, and other cardiometabolic disorders are common in patients with type 2 diabetes mellitus and are independently associated with chronic low-grade inflammation and elevated CBC-derived inflammatory indices, including NLR, PLR, and SII. Consequently, unmeasured differences in comorbidity burden between glycemic groups may have influenced the observed inflammatory profiles. Therefore, the net effect of unmeasured comorbidities on the study findings remains uncertain and should be considered when interpreting the results. Pharmacological attenuation of systemic inflammation deserves specific consideration. By definition, patients receiving glucose-lowering medication were classified as having diabetes irrespective of their concurrent glycemic values, and most of this group (82.8%) was ICD-coded and under clinical management, as reflected in its low median HbA1c (5.50%). Metformin, SGLT-2 inhibitors, DPP-4 inhibitors, and GLP-1 receptor agonists reduce CRP and other markers of systemic inflammation through effects that are not fully explained by improved glycemic control [37]. Consistent with this, CRP was approximately threefold lower in the ICD-coded than in the laboratory-defined subgroup. Treatment-related suppression of systemic inflammation is therefore a plausible contributor to the absence of significant differences in CRP, ESR, and CBC-derived indices between the diabetes and normoglycemia groups, although medication class, dose, and adherence could not be verified from our records.

The multivariable analyses provide the principal clinical message of the present study. Although patients with prediabetes and diabetes exhibited higher disability scores in the unadjusted analyses, glycemic status was no longer independently associated with disability as measured by the EDSS score after adjustment for age, sex, disease duration, MS phenotype, and DMT. Instead, older age, longer disease duration, and particularly progressive MS phenotype emerged as the dominant determinants of disability. These findings indicate that the apparent association between impaired glycemic status and disability is largely explained by established clinical characteristics rather than by glycemic dysregulation itself. Progressive MS phenotype showed the strongest contribution to disability, consistent with the well-recognized accumulation of irreversible neuroaxonal injury and progressive neurological impairment that characterize this disease stage [37,38]. Likewise, the independent effects of age and disease duration agree with numerous longitudinal studies demonstrating that disability accumulation in MS is primarily driven by aging and disease progression rather than isolated metabolic abnormalities [39,40,41,42]. Therefore, our findings suggest that impaired glycemic status should be considered a marker of a less favorable clinical and metabolic profile rather than an independent predictor of disability. This distinction is clinically important because it indicates that metabolic dysfunction accompanies disease progression but does not appear to independently determine neurological disability after accounting for major disease-related factors.

The multivariable analysis of relapse activity yielded findings similar to those observed for disability. After adjustment for demographic and disease-related characteristics, neither prediabetes nor diabetes was independently associated with ARR. Instead, older age, longer disease duration, and progressive MS phenotype were independently associated with lower ARR. These findings are biologically plausible because relapse frequency generally declines with advancing age and disease duration, while progressive forms of MS are characterized by a gradual accumulation of neurological disability with fewer overt inflammatory relapses [38,39,40,41,42]. Therefore, the inverse association between metabolic markers and ARR observed in the univariable analyses is likely attributable to differences in disease stage rather than a direct effect of impaired glycemic regulation. Collectively, these findings further support the conclusion that established clinical characteristics remain the primary determinants of relapse activity, whereas glycemic dysregulation appears to accompany, rather than independently influence, the clinical course of MS.

### Strengths and Limitations

This study has several limitations. First, its retrospective, single-center design limits the ability to establish causal relationships and may restrict the generalizability of the findings to other MS populations. Second, glycemic status was determined using routinely available clinical data, and longitudinal changes in glucose metabolism during follow-up could not be assessed. Third, information on lifestyle factors, including dietary habits, physical activity, and socioeconomic status, was unavailable and therefore could not be included in the analyses. Fourth, the study period (January 2020 to January 2025) overlapped with the COVID-19 pandemic and the subsequent COVID-19 vaccination period. Because of the retrospective study design, information regarding prior SARS-CoV-2 infection, disease severity, and vaccination status was not consistently available and therefore could not be incorporated into the analyses. Consequently, the potential influence of these factors on inflammatory and hematological parameters cannot be completely excluded. Fifth, detailed information regarding concomitant medications, including disease-modifying therapies, corticosteroids, lipid-lowering agents, and other drugs that may influence glycemic control, hematological parameters, or systemic inflammation, was not consistently available. Although glucose-lowering medications were considered in the classification of diabetes, the potential confounding effects of other medications could not be fully evaluated. Sixth, detailed information regarding comorbid conditions that may influence systemic inflammation or CBC-derived inflammatory indices, such as hypertension, cardiovascular disease, obesity, and other chronic metabolic disorders, was not consistently available. Although patients with acute infection, malignancy, chronic organ failure, and concomitant systemic inflammatory or autoimmune diseases were excluded, residual confounding related to unmeasured comorbidities cannot be completely excluded. Seventh, although several potential confounders were adjusted for, residual confounding from unmeasured variables cannot be completely excluded. Finally, the cross-sectional assessment of laboratory parameters precluded evaluation of the temporal relationship between metabolic dysfunction and disease progression. Moreover, the exclusion of patients who received corticosteroid treatment within the preceding three months may have introduced selection bias by preferentially excluding patients with recent inflammatory activity and a higher relapse risk, which may have attenuated the observed association between glycemic status and relapse activity. Therefore, these findings should be interpreted with appropriate caution.

Despite these limitations, this study has several important strengths. To our knowledge, this is one of the largest single-center studies to comprehensively evaluate the relationship between glycemic status and clinical outcomes in patients with MS. Unlike previous studies focusing primarily on diabetes prevalence or disability alone, we simultaneously assessed disability, relapse activity, inflammatory biomarkers, hematological inflammatory indices, lipid profile, and novel metabolic indices, including the TyG index and the AIP. Furthermore, the use of multivariable regression models with robust standard errors allowed adjustment for major clinical confounders, enabling differentiation between crude associations and independent determinants of disability and relapse activity. These findings provide a comprehensive overview of the interaction between glycemic dysregulation, metabolic burden, and clinical characteristics in MS and may serve as a foundation for future prospective multicenter studies.

Impaired glycemic status in patients with multiple sclerosis was associated with a less favorable metabolic and inflammatory profile, accompanied by higher disability and differences in clinical disease burden. However, after adjustment for major clinical characteristics, including age, disease duration, and MS phenotype, glycemic status was not independently associated with disability or relapse activity. These findings suggest that glycemic dysregulation accompanies a more adverse clinical and metabolic phenotype rather than acting as an independent determinant of disease severity. Instead, age, disease duration, and progressive MS phenotype appear to be the predominant factors underlying disability accumulation and relapse patterns in MS. Accordingly, glycemic abnormalities should be regarded as markers of an unfavorable metabolic and inflammatory state rather than direct drivers of neurological disability. Nevertheless, routine assessment of glycemic and metabolic status may provide valuable complementary information for comprehensive clinical evaluation and risk stratification in patients with MS. Future prospective longitudinal studies are warranted to clarify the temporal relationship between metabolic dysfunction and disease progression and to determine whether improving glycemic control can influence long-term clinical outcomes.

## Figures and Tables

**Table 1:** Demographic and clinical characteristics of patients according to glycemic status.

Variable	Normoglycemia (n = 241)	Prediabetes (n = 98)	Diabetes (n = 116)	*p* Value	Effect Size
Age, years	38.00 [29.00–47.00]	49.00 [38.00–57.00]	42.00 [36.75–54.00]	<0.001	0.083
Sex				0.156	0.090
Female	168 (69.7)	59 (60.2)	72 (62.1)		
Male	73 (30.3)	39 (39.8)	44 (37.9)		
Disease duration, years	7.80 [4.32–13.80]	12.22 [7.46–14.88]	8.16 [7.46–14.88]	0.005	0.019
MS phenotype				<0.001	0.149
RRMS	223 (92.5)	73 (74.5)	99 (85.3)		
SPMS	12 (5.0)	16 (16.3)	11 (9.5)		
PPMS	6 (2.5)	9 (9.2)	6 (5.2)		
DMT step				0.324	0.088
Step 1	101 (41.9)	45 (45.9)	49 (42.2)		
Step 2	111 (46.1)	42 (42.9)	50 (43.1)		
Step 3	14 (5.8)	6 (6.1)	14 (12.1)		
Step 4	15 (6.2)	5 (5.1)	3 (2.6)		
EDSS score	2.00 [1.50–2.00]	2.00 [1.50–3.00]	2.00 [1.50–2.00]	<0.001	0.039
EDSS category				<0.001	0.154
Mild, ≤3.0	225 (93.4)	75 (76.5)	97 (83.6)		
Moderate, 3.5–5.5	16 (6.6)	22 (22.4)	17 (14.7)		
Advanced, ≥6.0	0 (0.0)	1 (1.0)	2 (1.7)		
Cumulative number of relapses since disease onset	1.00 [1.00–1.00] (0–4)	1.00 [1.00–1.00] (0–3)	1.00 [1.00–1.00] (0–5)	0.314	0.001
Annualized relapse rate	0.13 [0.07–0.22]	0.08 [0.05–0.13]	0.13 [0.07–0.13]	0.005	0.019
ARR category				0.201	0.080
Relapse-free, 0	28 (11.6)	17 (17.3)	13 (11.2)		
Low, >0 to <0.5	211 (87.6)	81 (82.7)	100 (86.2)		
High, ≥0.5	2 (0.8)	0 (0.0)	3 (2.6)		

Note. ARR, annualized relapse rate; DMT, disease-modifying therapy; EDSS, Expanded Disability Status Scale; IQR, interquartile range; MS, multiple sclerosis; PPMS, primary progressive multiple sclerosis; RRMS, relapsing-remitting multiple sclerosis; SPMS, secondary progressive multiple sclerosis. Continuous variables are median [IQR]; cumulative relapse counts additionally include observed ranges. Categorical variables are n (%). ARR was calculated as cumulative documented relapses since disease onset divided by disease duration in years. DMT steps: Step 1, dimethyl fumarate, teriflunomide, glatiramer acetate, or interferon beta; Step 2, ocrelizumab, rituximab, or fingolimod; Step 3, natalizumab; Step 4, cladribine. Categorical variables were compared using Pearson’s chi-square test or the Fisher–Freeman–Halton exact test, as appropriate.

**Table 2:** Hematological and inflammatory markers according to glycemic status.

Variable	Normoglycemia (n = 241)	Prediabetes (n = 98)	Diabetes (n = 116)	Available n (N/P/D)	*p* Value	FDR q Value	ε^2^
WBC, ×10^9^/L	6.79 [5.59–8.15]	7.04 [5.49–8.72]	7.12 [5.89–8.62]	241/98/116	0.181	0.414	0.003
Neutrophil, ×10^9^/L	4.36 [3.33–5.38]	4.54 [3.66–5.77]	4.55 [3.62–5.69]	241/98/116	0.238	0.436	0.002
Lymphocyte, ×10^9^/L	1.76 [1.26–2.21]	1.76 [1.11–2.26]	1.75 [1.21–2.29]	241/98/116	0.857	0.857	0.0
Monocyte, ×10^9^/L	0.45 [0.36–0.54]	0.46 [0.38–0.56]	0.45 [0.38–0.54]	241/98/116	0.590	0.649	0.0
Platelet, ×10^9^/L	248.00 [210.00–281.00]	263.50 [223.50–296.00]	262.50 [226.75–302.25]	241/98/116	0.011	0.040	0.016
CRP, mg/L	1.32 [0.72–2.88]	2.43 [1.16–5.40]	1.74 [0.70–4.44]	241/98/116	<0.001	0.004	0.03
ESR, mm/h	6.00 [2.00–14.00]	10.00 [5.00–18.75]	9.00 [3.00–19.00]	241/98/116	0.002	0.013	0.022
NLR	2.62 [1.70–3.85]	2.58 [1.98–4.30]	2.53 [2.01–3.68]	241/98/116	0.404	0.529	0.0
MLR	0.26 [0.19–0.38]	0.27 [0.20–0.43]	0.26 [0.20–0.36]	241/98/116	0.391	0.529	0.0
PLR	138.87 [110.27–203.66]	150.19 [112.42–238.40]	157.31 [111.69–221.06]	241/98/116	0.433	0.529	0.0
SII	633.98 [413.24–967.05]	705.16 [460.52–1188.86]	675.00 [508.44–1011.85]	241/98/116	0.188	0.414	0.003

Note. CRP, C-reactive protein; ESR, erythrocyte sedimentation rate; MLR, monocyte-to-lymphocyte ratio; NLR, neutrophil-to-lymphocyte ratio; PLR, platelet-to-lymphocyte ratio; SII, systemic immune-inflammation index; WBC, white blood cell count. Variables are presented as median [interquartile range]. Glycemic status was categorized into normoglycemia, prediabetes, and diabetes. Patients with ICD-10 diabetes codes E11 and/or laboratory-defined diabetes were classified as the diabetes group. Kruskal–Wallis tests were used, with epsilon squared (ε^2^) as effect size.

**Table 3:** Metabolic, lipid, renal, and hepatic profile according to glycemic status.

Variable	Normoglycemia	Prediabetes	Diabetes	Available n (N/P/D)	*p* Value	FDR q Value	Effect Size
Glucose, mg/dL	85.00 [81.00–91.00]	99.00 [90.00–105.00]	89.00 [83.00–105.25]	241/98/116	<0.001	<0.001	0.178 (ε^2^)
HbA1c, %	5.20 [5.10–5.40]	5.80 [5.70–6.00]	5.50 [5.30–5.93]	240/98/116	<0.001	<0.001	0.310 (ε^2^)
Total cholesterol, mg/dL	198.00 [178.00–230.00]	222.50 [188.25–246.50]	211.50 [191.75–241.50]	241/98/116	<0.001	<0.001	0.028 (ε^2^)
LDL cholesterol, mg/dL	117.00 [99.00–140.00]	137.00 [114.25–158.75]	127.00 [110.50–149.00]	238/98/115	<0.001	<0.001	0.032 (ε^2^)
HDL cholesterol, mg/dL	59.00 [51.00–69.00]	54.50 [47.25–65.00]	56.00 [50.00–66.00]	241/98/116	0.034	0.040	0.011 (ε^2^)
Triglycerides, mg/dL	95.00 [68.00–136.00]	127.50 [81.50–174.75]	121.00 [78.75–170.25]	241/98/116	<0.001	<0.001	0.037 (ε^2^)
AIP	−0.15 ± 0.25	−0.01 ± 0.32	−0.04 ± 0.29	241/98/116	<0.001	<0.001	0.048 (η^2^)
TyG index	8.33 ± 0.51	8.70 ± 0.61	8.70 ± 0.73	241/98/116	<0.001	<0.001	0.091 (η^2^)
Creatinine, mg/dL	0.71 [0.60–0.82]	0.75 [0.60–0.85]	0.72 [0.61–0.80]	240/98/116	0.491	0.491	0.000 (ε^2^)
eGFR, mL/min/1.73 m^2^	112.00 [99.75–122.00]	104.00 [94.25–113.00]	109.00 [99.50–116.00]	240/98/116	<0.001	<0.001	0.032 (ε^2^)
AST, U/L	20.50 [17.00–25.00]	21.50 [18.00–26.00]	22.00 [18.00–26.00]	240/98/113	0.370	0.401	0.000 (ε^2^)
ALT, U/L	17.00 [14.00–26.00]	19.50 [16.00–28.75]	21.00 [16.00–30.00]	240/98/113	<0.001	<0.001	0.029 (ε^2^)
GGT, U/L	17.00 [13.00–27.00]	22.00 [17.00–33.00]	21.00 [15.00–32.50]	225/93/107	<0.001	<0.001	0.045 (ε^2^)

Note. AIP, atherogenic index of plasma; ALT, alanine aminotransferase; AST, aspartate aminotransferase; eGFR, estimated glomerular filtration rate; GGT, gamma-glutamyl transferase; HbA1c, glycated hemoglobin; HDL, high-density lipoprotein; LDL, low-density lipoprotein; TyG, triglyceride-glucose index. Non-normally distributed variables are median [interquartile range]; AIP and TyG are mean ± standard deviation. Kruskal–Wallis tests were used except AIP (one-way ANOVA) and TyG (Welch ANOVA). Benjamini–Hochberg correction was applied across 13 comparisons. Available n is shown for each group.

**Table 4:** Correlations of glycemic, metabolic, lipid, and inflammatory markers with clinical MS outcomes.

Marker	n	EDSS ρ	EDSS *p*	EDSS q	ARR ρ	ARR *p*	ARR q	Disease Duration ρ	Disease Duration *p*	Disease Duration q
Glucose, mg/dL	455	0.189	<0.001	<0.001	−0.198	<0.001	<0.001	0.201	<0.001	<0.001
HbA1c, %	454	0.249	<0.001	<0.001	−0.264	<0.001	<0.001	0.272	<0.001	<0.001
TyG index	455	0.227	<0.001	<0.001	−0.236	<0.001	<0.001	0.287	<0.001	<0.001
AIP	455	0.14	0.003	0.005	−0.168	<0.001	<0.001	0.206	<0.001	<0.001
Total cholesterol, mg/dL	455	0.211	<0.001	<0.001	−0.294	<0.001	<0.001	0.298	<0.001	<0.001
LDL cholesterol, mg/dL	451	0.181	<0.001	<0.001	−0.298	<0.001	<0.001	0.281	<0.001	<0.001
HDL cholesterol, mg/dL	455	0.041	0.387	0.452	−0.011	0.822	0.842	0.021	0.661	0.712
Triglycerides, mg/dL	455	0.187	<0.001	<0.001	−0.21	<0.001	<0.001	0.254	<0.001	<0.001
CRP, mg/L	455	0.163	<0.001	<0.001	−0.117	0.012	0.019	0.132	0.005	0.008
ESR, mm/h	455	0.177	<0.001	<0.001	−0.133	0.005	0.008	0.125	0.008	0.012
NLR	455	0.075	0.112	0.163	0.077	0.103	0.154	0.005	0.920	0.920
MLR	455	0.053	0.256	0.326	0.062	0.188	0.247	0.052	0.270	0.333
PLR	455	0.021	0.660	0.712	0.05	0.283	0.340	0.026	0.575	0.653
SII	455	0.073	0.121	0.170	0.071	0.133	0.180	−0.011	0.821	0.842

Note. AIP, atherogenic index of plasma; ARR, annualized relapse rate; CRP, C-reactive protein; EDSS, Expanded Disability Status Scale; ESR, erythrocyte sedimentation rate; HbA1c, glycated hemoglobin; HDL, high-density lipoprotein; LDL, low-density lipoprotein; MLR, monocyte-to-lymphocyte ratio; MS, multiple sclerosis; NLR, neutrophil-to-lymphocyte ratio; PLR, platelet-to-lymphocyte ratio; SII, systemic immune-inflammation index; TyG, triglyceride-glucose index. Correlations were assessed using Spearman correlation analysis. Correlation coefficients are presented as Spearman’s rho (ρ), which also represents the effect size. Benjamini–Hochberg correction was applied across all 42 correlations. Positive ρ values indicate a positive association, whereas negative ρ values indicate an inverse association. ᶠSpearman correlation test. Available sample size varied slightly across variables due to missing laboratory records.

**Table 5:** Multivariable linear regression models for EDSS and ARR.

Predictor	EDSS, B [95% CI]	EDSS Standardized β [95% CI]	*p* Value	ARR, B [95% CI]	ARR Standardized β [95% CI]	*p* Value	VIF	Tolerance
Prediabetes vs. normoglycemia	0.039 [−0.105 to 0.183]	0.018 [−0.048 to 0.083]	0.592	0.002 [−0.016 to 0.021]	0.008 [−0.053 to 0.069]	0.792	1.230	0.813
Diabetes vs. normoglycemia	0.084 [−0.049 to 0.217]	0.040 [−0.024 to 0.104]	0.217	0.026 [−0.005 to 0.057]	0.091 [−0.018 to 0.200]	0.103	1.154	0.867
Age, per year	0.015 [0.005 to 0.024]	0.191 [0.065 to 0.316]	0.003	−0.003 [−0.004 to −0.002]	−0.293 [−0.419 to −0.167]	<0.001	2.040	0.490
Male sex	−0.127 [−0.256 to 0.002]	−0.067 [−0.134 to 0.001]	0.053	−0.004 [−0.027 to 0.019]	−0.015 [−0.103 to 0.074]	0.748	1.115	0.897
Disease duration, per year	0.027 [0.005 to 0.049]	0.167 [0.030 to 0.303]	0.017	−0.006 [−0.009 to −0.003]	−0.286 [−0.416 to −0.156]	<0.001	1.800	0.556
Progressive MS phenotype	1.708 [1.392 to 2.024]	0.639 [0.521 to 0.757]	<0.001	−0.027 [−0.050 to −0.003]	−0.073 [−0.137 to −0.009]	0.026	1.279	0.782
DMT step	−0.028 [−0.089 to 0.032]	−0.025 [−0.078 to 0.028]	0.356	−0.007 [−0.019 to 0.004]	−0.047 [−0.119 to 0.025]	0.200	1.014	0.986

Note. ARR, annualized relapse rate; CI, confidence interval; DMT, disease-modifying therapy; EDSS, Expanded Disability Status Scale; VIF, variance inflation factor. Multivariable linear regression models were estimated using HC3 heteroscedasticity-robust standard errors. Normoglycemia, female sex, and relapsing-remitting MS were the reference categories; progressive MS phenotype included secondary and primary progressive MS. Unstandardized B and standardized β coefficients are presented with 95% confidence intervals. No substantial multicollinearity was detected (all VIF < 5 and tolerance > 0.20). Model diagnostics: EDSS model, R^2^ = 0.645, adjusted R^2^ = 0.639, F = 76.29, Durbin-Watson = 1.942, Breusch-Pagan *p* < 0.001; ARR model, R^2^ = 0.308, adjusted R^2^ = 0.297, F = 24.50, Durbin-Watson = 1.997, Breusch-Pagan *p* < 0.001.

## Data Availability

The data supporting the findings of this study are available from the corresponding author upon reasonable request.

## Supplementary Materials

The following supporting information can be downloaded at: https://www.mdpi.com/article/10.3390/jcm15155960/s1, Supplementary Table S1. Pairwise post-hoc comparisons for variables showing significant differences across glycemic-status groups; Supplementary Table S2. Extended laboratory profile according to glycemic status; Supplementary Table S3. Missing-data profile of variables included in the main analyses; Supplementary Table S4. Diabetes classification, HbA1c distribution, and clinical/metabolic characteristics of diabetes subgroups, Panel A. Basis of diabetes classification and HbA1c distribution, Panel B. Clinical and metabolic comparison of ICD-coded and laboratory-defined diabetes; Supplementary Table S5. Sensitivity analyses of glycemic-status associations with EDSS score and annualized relapse rate.
