# Supplementary material for: Association of Glycemic Status with Disability, Relapse Activity, Inflammatory Markers, and Metabolic Profile in Patients with Multiple Sclerosis
Source: J Clin Med. 2026 Jul 30;15(15):5960. doi: 10.3390/jcm15155960 (PMC13466910; doi:10.3390/jcm15155960)
Supplement: Supplementary file 1 [file jcm-15-05960-s001.zip › jcm-4432368-supplementary.pdf]

SUPPLEMENTARY MATERIAL

Supplementary Table S1. Pairwise post-hoc comparisons for variables showing significant differences across glycemic-status groups

Variable	Comparison	Post-hoc test	Adjusted p value	Effect size
Age, years	Normoglycemia vs Prediabetes	Dunn-Bonferroni	<0.001	0.278
Age, years	Normoglycemia vs Diabetes	Dunn-Bonferroni	<0.001	0.177
Age, years	Prediabetes vs Diabetes	Dunn-Bonferroni	0.117	0.097
Disease duration, years	Normoglycemia vs Prediabetes	Dunn-Bonferroni	0.005	0.147
Disease duration, years	Normoglycemia vs Diabetes	Dunn-Bonferroni	0.219	0.084
Disease duration, years	Prediabetes vs Diabetes	Dunn-Bonferroni	0.615	0.059
EDSS score	Normoglycemia vs Prediabetes	Dunn-Bonferroni	<0.001	0.200
EDSS score	Normoglycemia vs Diabetes	Dunn-Bonferroni	0.048	0.113
EDSS score	Prediabetes vs Diabetes	Dunn-Bonferroni	0.242	0.082
Annualized relapse rate	Normoglycemia vs Prediabetes	Dunn-Bonferroni	0.003	0.152
Annualized relapse rate	Normoglycemia vs Diabetes	Dunn-Bonferroni	1.000	0.036
Annualized relapse rate	Prediabetes vs Diabetes	Dunn-Bonferroni	0.083	0.103
Platelet count	Normoglycemia vs Prediabetes	Dunn-Bonferroni	0.114	0.097
Platelet count	Normoglycemia vs Diabetes	Dunn-Bonferroni	0.020	0.127
Platelet count	Prediabetes vs Diabetes	Dunn-Bonferroni	1.000	0.020
CRP	Normoglycemia vs Prediabetes	Dunn-Bonferroni	<0.001	0.184
CRP	Normoglycemia vs Diabetes	Dunn-Bonferroni	0.250	0.081
CRP	Prediabetes vs Diabetes	Dunn-Bonferroni	0.135	0.094
ESR	Normoglycemia vs Prediabetes	Dunn-Bonferroni	0.005	0.149
ESR	Normoglycemia vs Diabetes	Dunn-Bonferroni	0.059	0.109
ESR	Prediabetes vs Diabetes	Dunn-Bonferroni	1.000	0.040
Glucose, mg/dL	Normoglycemia vs Prediabetes	Dunn-Bonferroni	<0.001	0.414
Glucose, mg/dL	Normoglycemia vs Diabetes	Dunn-Bonferroni	<0.001	0.220
Glucose, mg/dL	Prediabetes vs Diabetes	Dunn-Bonferroni	<0.001	0.180
HbA1c, %	Normoglycemia vs Prediabetes	Dunn-Bonferroni	<0.001	0.524
HbA1c, %	Normoglycemia vs Diabetes	Dunn-Bonferroni	<0.001	0.344
HbA1c, %	Prediabetes vs Diabetes	Dunn-Bonferroni	<0.001	0.174
Total cholesterol, mg/dL	Normoglycemia vs Prediabetes	Dunn-Bonferroni	0.001	0.165
Total cholesterol, mg/dL	Normoglycemia vs Diabetes	Dunn-Bonferroni	0.035	0.118
Total cholesterol, mg/dL	Prediabetes vs Diabetes	Dunn-Bonferroni	0.966	0.046
LDL cholesterol, mg/dL	Normoglycemia vs Prediabetes	Dunn-Bonferroni	<0.001	0.179
LDL cholesterol, mg/dL	Normoglycemia vs Diabetes	Dunn-Bonferroni	0.044	0.115
LDL cholesterol, mg/dL	Prediabetes vs Diabetes	Dunn-Bonferroni	0.586	0.061
HDL cholesterol, mg/dL	Normoglycemia vs Prediabetes	Dunn-Bonferroni	0.042	0.115
HDL cholesterol, mg/dL	Normoglycemia vs Diabetes	Dunn-Bonferroni	0.345	0.074
HDL cholesterol, mg/dL	Prediabetes vs Diabetes	Dunn-Bonferroni	1.000	0.040
Triglycerides, mg/dL	Normoglycemia vs Prediabetes	Dunn-Bonferroni	<0.001	0.177
Triglycerides, mg/dL	Normoglycemia vs Diabetes	Dunn-Bonferroni	0.004	0.151
Triglycerides, mg/dL	Prediabetes vs Diabetes	Dunn-Bonferroni	1.000	0.031
eGFR, mL/min/1.73 m2	Normoglycemia vs Prediabetes	Dunn-Bonferroni	<0.001	0.189

Variable	Comparison	Post-hoc test	Adjusted p value	Effect size
eGFR, mL/min/1.73 m2	Normoglycemia vs Diabetes	Dunn-Bonferroni	0.221	0.084
eGFR, mL/min/1.73 m2	Prediabetes vs Diabetes	Dunn-Bonferroni	0.123	0.096
ALT, U/L	Normoglycemia vs Prediabetes	Dunn-Bonferroni	0.016	0.131
ALT, U/L	Normoglycemia vs Diabetes	Dunn-Bonferroni	0.002	0.161
ALT, U/L	Prediabetes vs Diabetes	Dunn-Bonferroni	1.000	0.019
GGT, U/L	Normoglycemia vs Prediabetes	Dunn-Bonferroni	<0.001	0.211
GGT, U/L	Normoglycemia vs Diabetes	Dunn-Bonferroni	0.025	0.128
GGT, U/L	Prediabetes vs Diabetes	Dunn-Bonferroni	0.334	0.077
AIP	Diabetes vs Normoglycemia	Tukey	0.002	0.402
AIP	Diabetes vs Prediabetes	Tukey	0.644	0.112
AIP	Normoglycemia vs Prediabetes	Tukey	<0.001	0.516
TyG index	Normoglycemia vs Prediabetes	Welch pairwise with Bonferroni	<0.001	0.699
TyG index	Normoglycemia vs Diabetes	Welch pairwise with Bonferroni	<0.001	0.628
TyG index	Prediabetes vs Diabetes	Welch pairwise with Bonferroni	1.000	0.009
MS phenotype	Normoglycemia vs Prediabetes	Fisher–Freeman–Halton exact with Bonferroni	<0.001	0.246
MS phenotype	Normoglycemia vs Diabetes	Fisher–Freeman–Halton exact with Bonferroni	0.271	0.113
MS phenotype	Prediabetes vs Diabetes	Fisher–Freeman–Halton exact with Bonferroni	0.426	0.136
EDSS category	Normoglycemia vs Prediabetes	Fisher–Freeman–Halton exact with Bonferroni	<0.001	0.244
EDSS category	Normoglycemia vs Diabetes	Fisher–Freeman–Halton exact with Bonferroni	0.011	0.171
EDSS category	Prediabetes vs Diabetes	Fisher–Freeman–Halton exact with Bonferroni	1.000	0.103

Note. Dunn–Bonferroni post-hoc tests were used after Kruskal–Wallis analyses. AIP used Tukey comparisons; TyG used Welch pairwise comparisons with Bonferroni adjustment. Categorical pairwise comparisons used Fisher–Freeman–Halton exact tests with Bonferroni correction. Effect sizes are r, Hedges' g, or Cramér's V, as appropriate.

Supplementary Table S2. Extended laboratory profile according to glycemic status

Variable	Valid n	Normoglycemia (n=241)	Prediabetes (n=98)	Diabetes (n=116)	p value	FDR q value	Effect size
RBC $\times 10^{12}$/L	455	4.64 [4.42-4.95]	4.77 [4.45-5.01]	4.70 [4.42-5.00]	0.340[a]	0.592	0.000
Hemoglobin, g/L	455	137.00 [127.00-147.00]	135.50 [125.00-147.50]	136.50 [126.00-146.00]	0.761[a]	0.893	0.000
Hematocrit, %	455	41.37 ± 4.07	41.16 ± 4.39	41.37 ± 4.23	0.906[d]	0.906	0.000
MCV, fL	455	88.40 [85.30-92.00]	87.15 [83.43-90.40]	88.40 [85.38-92.50]	0.031[a]	0.103	0.011
MCH, pg	455	29.50 [28.10-30.50]	29.00 [27.38-29.98]	29.30 [28.00-30.32]	0.026[a]	0.095	0.012
MCHC, g/L	455	330.00 [324.00-337.00]	330.00 [322.00-336.00]	328.00 [322.00-336.00]	0.270[a]	0.577	0.001
RDW, %	455	13.30 [12.80-13.90]	13.50 [13.10-14.30]	13.45 [12.80-14.00]	0.014[a]	0.093	0.014
MPV, fL	455	9.60 [9.10-10.20]	9.65 [9.00-10.60]	9.60 [9.07-10.20]	0.798[a]	0.893	0.000
Plateletcrit %	455	0.24 [0.21-0.27]	0.25 [0.23-0.29]	0.25 [0.22-0.28]	0.005[a]	0.064	0.019
PDW	455	16.08 ± 0.36	16.06 ± 0.37	16.04 ± 0.37	0.588[d]	0.790	0.000
Neutrophil, %	455	64.90 [57.60-72.00]	64.30 [59.23-73.40]	65.00 [60.20-71.93]	0.564[a]	0.790	0.000
Lymphocyte, %	455	25.51 ± 9.41	23.79 ± 9.25	25.37 ± 9.57	0.294[d]	0.577	0.001
Monocyte, %	455	6.60 [5.50-8.20]	6.50 [5.50-8.12]	6.60 [5.60-7.62]	0.732[a]	0.893	0.000
Eosinophil count	455	0.12 [0.07-0.20]	0.13 [0.08-0.21]	0.14 [0.08-0.20]	0.886[a]	0.906	0.000
Eosinophil, %	455	1.90 [1.10-3.10]	2.05 [1.20-2.80]	1.90 [1.10-2.90]	0.852[a]	0.906	0.000
Basophil count	455	0.03 [0.02-0.04]	0.03 [0.02-0.04]	0.03 [0.02-0.04]	0.427[a]	0.627	0.000
Basophil, %	455	0.50 [0.30-0.60]	0.40 [0.30-0.60]	0.40 [0.30-0.60]	0.077[a]	0.202	0.007
BUN, mg/dL	452	12.00 [10.00-14.00]	13.00 [11.00-15.00]	13.00 [10.25-15.00]	0.022[a]	0.095	0.012
Urea, mg/dL	452	26.00 [21.00-30.00]	27.50 [24.00-33.00]	27.00 [22.25-33.00]	0.019[a]	0.093	0.013
Uric acid, mg/dL	455	4.20 [3.90-5.40]	5.10 [4.20-5.65]	4.40 [3.90-5.30]	0.103[a]	0.242	0.006
Sodium, mmol/L	454	139.00 [138.00-140.00]	139.00 [138.00-140.00]	139.00 [137.75-140.00]	0.061[a]	0.180	0.008
Potassium, mmol/L	454	4.34 [4.13-4.55]	4.38 [4.10-4.56]	4.36 [4.13-4.61]	0.614[a]	0.802	0.000
Chloride, mmol/L	216	104.00 [103.00-106.00]	105.00 [103.00-106.00]	104.00 [102.00-105.00]	0.020[a]	0.093	0.027
Calcium, mg/dL	454	9.73 [9.48-9.94]	9.58 [9.31-9.93]	9.66 [9.42-9.99]	0.308[a]	0.580	0.001
Phosphorus, mg/dL	218	3.40 ± 0.54	3.49 ± 0.63	3.43 ± 0.48	0.650[d]	0.826	0.000
Magnesium, mg/dL	223	2.01 ± 0.13	1.99 ± 0.15	2.00 ± 0.15	0.772[d]	0.893	0.000
ALP, U/L	170	59.00 [49.00-71.50]	71.50 [59.25-88.00]	65.00 [50.50-77.00]	0.002[a]	0.054	0.061
LDH, U/L	455	156.00 [150.00-163.00]	156.50 [150.00-169.00]	156.00 [150.00-172.00]	0.055[a]	0.173	0.008
CK, U/L	201	82.00 [61.00-108.00]	83.00 [63.00-145.00]	62.00 [47.00-99.00]	0.017[a]	0.093	0.031
Total protein, g/L	182	71.85 ± 5.13	71.04 ± 4.35	72.49 ± 5.11	0.409[d]	0.620	0.000
Albumin, g/L	455	44.00 [43.00-46.00]	44.00 [43.00-46.00]	44.00 [43.00-46.00]	0.019[a]	0.093	0.013
Globulin, g/L	167	27.00 [24.00-30.00]	28.00 [25.00-31.50]	28.50 [25.75-31.00]	0.329[a]	0.592	0.001
Total bilirubin, mg/dL	199	0.56 [0.44-0.85]	0.52 [0.41-0.62]	0.51 [0.45-0.62]	0.286[a]	0.577	0.003
Direct bilirubin, mg/dL	195	0.11 [0.08-0.17]	0.10 [0.08-0.13]	0.11 [0.09-0.15]	0.114[a]	0.255	0.012

Variable	Valid n	Normoglycemia (n=241)	Prediabetes (n=98)	Diabetes (n=116)	p value	FDR q value	Effect size
Indirect bilirubin, mg/dL	150	0.46 [0.35-0.65]	0.43 [0.30-0.51]	0.40 [0.27-0.54]	0.068[a]	0.187	0.023
Amylase, U/L	98	76.21 ± 23.92	74.72 ± 24.69	73.37 ± 25.08	0.898[d]	0.906	0.000
Lipase, U/L	95	23.00 [15.00-32.25]	23.50 [18.50-29.75]	21.00 [15.50-26.00]	0.574[a]	0.790	0.000
Iron, µg/dL	199	91.00 [69.00-118.00]	63.00 [46.00-87.50]	80.00 [55.50-108.50]	<0.001[a]	0.016	0.071
Total iron-binding capacity, µg/dL	189	341.50 [310.00-372.00]	353.00 [315.25-400.25]	347.00 [307.00-381.00]	0.386[a]	0.605	0.000
UIBC, µg/dL	189	248.88 ± 68.37	288.83 ± 66.64	265.74 ± 71.15	0.005[d]	0.064	0.045
Ferritin, ng/mL	401	24.60 [13.55-55.25]	29.10 [11.45-68.30]	30.30 [13.85-64.10]	0.784[a]	0.893	0.000
Vitamin B12, pg/mL	420	329.00 [256.50-446.00]	343.00 [238.25-457.00]	354.00 [259.50-500.00]	0.373[a]	0.604	0.000
Folate, ng/mL	110	7.95 [5.38-11.65]	7.05 [5.24-10.95]	5.85 [4.33-7.55]	0.025[a]	0.095	0.050
25-hydroxy vitamin D, ng/mL	454	26.10 [18.27-37.62]	24.75 [18.35-33.25]	24.55 [16.77-32.42]	0.368[a]	0.604	0.000
TSH, mIU/L	455	1.65 [1.23-2.32]	1.67 [1.19-2.62]	1.58 [1.27-2.29]	0.845[a]	0.906	0.000
Free T3, pg/mL	455	3.17 [3.02-3.34]	3.17 [3.02-3.31]	3.17 [3.02-3.44]	0.102[a]	0.242	0.006
Free T4, ng/dL	455	0.82 [0.75-0.92]	0.79 [0.73-0.88]	0.84 [0.78-0.94]	0.014[a]	0.093	0.015

Note. Variables are presented as median [interquartile range] or mean ± standard deviation according to distribution. Benjamini–Hochberg FDR correction was applied across all 47 exploratory laboratory comparisons; q values are provided. These analyses are secondary and hypothesis-generating.

Supplementary Table S3. Missing-data profile of variables included in the main analyses

Variable	Norm valid	Norm missing	Prediab valid	Prediab missing	Diabetes valid	Diabetes missing	Total valid	Total missing	Missing (%)
HbA1c, %	240	1	98	0	116	0	454	1	0.2
Creatinine, mg/dL	240	1	98	0	116	0	454	1	0.2
eGFR, mL/min/1.73 m2	240	1	98	0	116	0	454	1	0.2
LDL cholesterol, mg/dL	238	3	98	0	115	1	451	4	0.9
AST, U/L	240	1	98	0	113	3	451	4	0.9
ALT, U/L	240	1	98	0	113	3	451	4	0.9
GGT, U/L	225	16	93	5	107	9	425	30	6.6

Note. Missingness was calculated within the protocol-defined analytic cohort (n=455). Outcome variables and model covariates were complete. Available-case analysis was used for occasional missing secondary laboratory values.

Supplementary Table S4. Diabetes classification, HbA1c distribution, and clinical/metabolic characteristics of diabetes subgroups

Panel A. Basis of diabetes classification and HbA1c distribution

Characteristic	Overall diabetes (n = 116)	ICD-coded diabetes (n = 96)	Laboratory-defined diabetes (n = 20)
Classification basis, n (%)	116 (100.0)	96 (82.8)	20 (17.2)
HbA1c, mean ± SD, %	5.93 ± 1.35	5.63 ± 0.83	7.40 ± 2.20
HbA1c, median [IQR], %	5.50 [5.30–5.93]	5.40 [5.20–5.80]	6.80 [5.75–8.50]
HbA1c, minimum–maximum, %	4.20–13.30	4.20–10.80	5.10–13.30
Met current laboratory diabetes threshold*, n (%)	30 (25.9%)	10 (10.4%)	20 (100.0%)
Did not meet current laboratory threshold*, n (%)	86 (74.1%)	86 (89.6%)	0 (0.0%)

Panel B. Clinical and metabolic comparison of ICD-coded and laboratory-defined diabetes

Variable	ICD-coded diabetes (n = 96)	Laboratory-defined diabetes (n = 20)	p value	FDR q value	Effect size
Age, years	42.00 [35.50–52.25]	53.00 [43.25–60.25]	0.016	0.040	-0.344 (r_rb)
Disease duration, years	7.93 [7.44–14.51]	12.84 [8.78–16.00]	0.007	0.023	-0.382 (r_rb)
EDSS score	2.00 [1.50–2.00]	2.00 [2.00–3.62]	0.025	0.057	-0.303 (r_rb)
Cumulative number of relapses	1.00 [1.00–1.00]	1.00 [1.00–1.00]	0.943	0.986	-0.008 (r_rb)
Annualized relapse rate	0.13 [0.07–0.15]	0.08 [0.06–0.12]	0.041	0.073	0.290 (r_rb)
Glucose, mg/dL	87.00 [82.00–96.00]	161.50 [125.50–214.50]	<0.001	<0.001	-0.754 (r_rb)
HbA1c, %	5.40 [5.20–5.80]	6.80 [5.75–8.50]	<0.001	<0.001	-0.601 (r_rb)
TyG index	8.52 [8.06–8.99]	9.39 [9.03–9.63]	<0.001	<0.001	-0.595 (r_rb)
AIP	-0.09 [-0.27–0.11]	0.12 [-0.02–0.17]	0.012	0.035	-0.358 (r_rb)
Total cholesterol, mg/dL	211.50 [191.75–241.50]	211.50 [193.00–228.25]	0.991	0.991	0.002 (r_rb)
LDL cholesterol, mg/dL (n = 95/20)	126.00 [110.00–149.50]	137.00 [118.75–149.00]	0.362	0.463	-0.131 (r_rb)
HDL cholesterol, mg/dL	58.50 [51.00–67.50]	48.00 [45.75–52.50]	<0.001	0.002	0.511 (r_rb)
Triglycerides, mg/dL	109.00 [75.00–170.25]	141.00 [99.50–172.25]	0.097	0.149	-0.237 (r_rb)
CRP, mg/L	1.67 [0.61–3.41]	4.63 [1.70–7.23]	0.006	0.023	-0.390 (r_rb)
ESR, mm/h	9.00 [3.00–17.25]	9.00 [3.00–20.75]	0.735	0.806	-0.048 (r_rb)
eGFR, mL/min/1.73 m²	109.50 [101.00–117.00]	104.00 [91.00–111.00]	0.034	0.066	0.302 (r_rb)
ALT, U/L (n = 93/20)	21.00 [16.00–29.00]	23.00 [16.00–44.75]	0.335	0.453	-0.138 (r_rb)
GGT, U/L (n = 88/19)	19.00 [13.75–32.00]	25.00 [20.50–44.00]	0.027	0.057	-0.325 (r_rb)
Sex, female/male	61 (63.5) / 35 (36.5)	11 (55.0) / 9 (45.0)	0.613	0.705	0.066 (V)
MS phenotype, RRMS/SPMS/PPMS	84 (87.5) / 7 (7.3) / 5 (5.2)	15 (75.0) / 4 (20.0) / 1 (5.0)	0.212	0.304	0.164 (V)
DMT step, 1/2/3/4	45 (46.9) / 40 (41.7) / 9 (9.4) / 2 (2.1)	4 (20.0) / 10 (50.0) / 5 (25.0) / 1 (5.0)	0.072	0.119	0.245 (V)
EDSS category, mild/moderate/advanced	85 (88.5) / 9 (9.4) / 2 (2.1)	12 (60.0) / 8 (40.0) / 0 (0.0)	0.004	0.018	0.330 (V)

Variable	ICD-coded diabetes (n = 96)	Laboratory-defined diabetes (n = 20)	p value	FDR q value	Effect size
ARR category, relapse-free/low/high	12 (12.5) / 81 (84.4) / 3 (3.1)	1 (5.0) / 19 (95.0) / 0 (0.0)	0.418	0.506	0.120 (V)

Note. *Current laboratory diabetes threshold was defined as fasting plasma glucose ≥126 mg/dL and/or HbA1c ≥6.5%. Among the 116 participants in the diabetes group, 96 (82.8%) had a documented ICD-10 diabetes diagnosis (E10–E14), whereas 20 (17.2%) were identified solely from laboratory criteria. Medication-use data were not reliably available; therefore, the ICD-coded subgroup could not be validly subdivided according to pharmacological treatment. Continuous subgroup comparisons used Mann–Whitney U tests and categorical comparisons used Fisher exact or Fisher–Freeman–Halton exact tests, as appropriate. Benjamini–Hochberg FDR correction was applied across the subgroup comparisons.

Supplementary Table S5. Sensitivity analyses of glycemic-status associations with EDSS score and annualized relapse rate

Analysis / predictor	EDSS B [95% CI]	EDSS standardized β [95% CI]	p value	ARR B [95% CI]	ARR standardized β [95% CI]	p value
Part A1. Four-category glycemic-status model (n = 455)	Part A1. Four-category glycemic-status model (n = 455)	Part A1. Four-category glycemic-status model (n = 455)	Part A1. Four-category glycemic-status model (n = 455)	Part A1. Four-category glycemic-status model (n = 455)	Part A1. Four-category glycemic-status model (n = 455)	Part A1. Four-category glycemic-status model (n = 455)
Prediabetes vs normoglycemia	0.044 [-0.100 to 0.188]	0.020 [-0.045 to 0.085]	0.550	0.002 [-0.016 to 0.021]	0.008 [-0.053 to 0.069]	0.803
ICD-coded diabetes vs normoglycemia	0.042 [-0.107 to 0.191]	0.019 [-0.048 to 0.086]	0.580	0.027 [-0.008 to 0.062]	0.089 [-0.025 to 0.203]	0.127
Laboratory-defined diabetes vs normoglycemia	0.301 [0.036 to 0.565]	0.068 [0.008 to 0.128]	0.026	0.019 [-0.003 to 0.042]	0.032 [-0.005 to 0.069]	0.092
Part A2. Model excluding laboratory-defined diabetes (n = 435)	Part A2. Model excluding laboratory-defined diabetes (n = 435)	Part A2. Model excluding laboratory-defined diabetes (n = 435)	Part A2. Model excluding laboratory-defined diabetes (n = 435)	Part A2. Model excluding laboratory-defined diabetes (n = 435)	Part A2. Model excluding laboratory-defined diabetes (n = 435)	Part A2. Model excluding laboratory-defined diabetes (n = 435)
Prediabetes vs normoglycemia	0.057 [-0.088 to 0.201]	0.027 [-0.042 to 0.096]	0.440	0.004 [-0.015 to 0.022]	0.012 [-0.050 to 0.073]	0.711
ICD-coded diabetes vs normoglycemia	0.048 [-0.101 to 0.197]	0.023 [-0.048 to 0.093]	0.530	0.028 [-0.007 to 0.063]	0.091 [-0.024 to 0.205]	0.121
Part B. DMT efficacy-category sensitivity model (n = 455; low efficacy n = 195; high efficacy n = 260)	Part B. DMT efficacy-category sensitivity model (n = 455; low efficacy n = 195; high efficacy n = 260)	Part B. DMT efficacy-category sensitivity model (n = 455; low efficacy n = 195; high efficacy n = 260)	Part B. DMT efficacy-category sensitivity model (n = 455; low efficacy n = 195; high efficacy n = 260)	Part B. DMT efficacy-category sensitivity model (n = 455; low efficacy n = 195; high efficacy n = 260)	Part B. DMT efficacy-category sensitivity model (n = 455; low efficacy n = 195; high efficacy n = 260)	Part B. DMT efficacy-category sensitivity model (n = 455; low efficacy n = 195; high efficacy n = 260)
Prediabetes vs normoglycemia	0.040 [-0.104 to 0.184]	0.018 [-0.047 to 0.084]	0.588	0.002 [-0.016 to 0.021]	0.008 [-0.053 to 0.069]	0.802
Diabetes vs normoglycemia	0.083 [-0.049 to 0.216]	0.040 [-0.024 to 0.104]	0.218	0.026 [-0.005 to 0.057]	0.091 [-0.019 to 0.200]	0.104
High-efficacy vs low-efficacy DMT	-0.001 [-0.107 to 0.105]	-0.000 [-0.059 to 0.058]	0.987	-0.010 [-0.031 to 0.010]	-0.041 [-0.122 to 0.039]	0.314

Note. All models used HC3 heteroscedasticity-consistent robust standard errors. Part A evaluated diabetes-definition sensitivity. Part B replaced ordinal DMT step with low- versus high-efficacy DMT. Normoglycemia, female sex, RRMS, and low-efficacy DMT were reference categories.
